# Case report: First Chinese patient with family partial lipodystrophy type 6 due to novel compound heterozygous mutations in the LIPE gene

**DOI:** 10.3389/fgene.2024.1417613

**Published:** 2024-07-24

**Authors:** Yimeng Zhou, Lin Zhang, Yang Ding, Yongzhen Zhai

**Affiliations:** Department of Infectious Diseases, Shengjing Hospital of China Medical University, Shenyang, China

**Keywords:** case report, family partial lipodystrophy, diabetes mellitus, LIPE, metabolic disorder, hepatic steatosis

## Abstract

**Background:**

Family partial lipodystrophy (FPLD) is a rare autosomal dominant disease characterized by disorders of variable body fat loss associated with metabolic complications. FPLD6 has only been reported in a limited number of cases. Here, we reported a Chinese FPLD6 patient with compound heterozygous mutations in the lipase E, hormone-sensitive type (*LIPE*) gene.

**Case presentation:**

A 20-year-old female patient presented with hypertriglyceridemia, diabetes mellitus, hepatomegaly, and hepatic steatosis. Subcutaneous fat was significantly diminished in her face, abdomen, and limbs. The patient was assessed by detailed clinical and biochemical examinations. A liver biopsy showed severe lipodystrophy. In addition, there were retinal changes, peripheral nerve damage, and renal tubular injury. Sequencing was performed on extracted DNA. Genetic analysis revealed that the patient had compound heterozygous mutations in the *LIPE* gene: c.2497_250ldel (p.Glu833LysfsTer22) and c.2705del (p.Ser902ThrfsTer27) heterozygous mutations. Verification revealed that this mutation was inherited from her father and mother, respectively, and that they formed newly discovered compound heterozygous mutations occurring in the *LIPE* gene, causing FPLD6.

**Conclusion:**

We reported the first case of FPLD6 in China. Gene analysis demonstrated compound heterozygous mutations in LIPE in this patient. Our case emphasizes the importance of genetic testing in young patients with severe metabolic syndromes.

## 1 Introduction

Family partial lipodystrophy (FPLD) is a rare hereditary disease, with an estimated prevalence of one in every one million people in the general population, although its prevalence may be underestimated (Rutkowska et al., 2022). Its characteristics include a reduction of subcutaneous fat tissue from peripheral fat storage areas, and the accumulation of fat tissue in other body areas, such as the face, neck, perineum, and abdominal cavity. These patients have normal fat distribution in childhood, followed by progressive and variant subcutaneous fat reduction in late childhood or around adolescence, usually starting from the limbs (leading to prominent muscular tissue), but changes can also be observed in the anterior abdomen and chest. Clinically, it is difficult to detect the subtle fat reduction in these patients, and due to a cushingoid appearance, many cases may be confused with Cushing’s syndrome. This disease is accompanied by metabolic abnormalities, including insulin resistance (IR), diabetes mellitus (DM), and dyslipidemia (Akinci et al., 2017; Bagias et al., 2020). Based on different pathogenic genes, there are six main types of this disease (FPLD1 to FPLD6), among which FPLD2 and FPLD3 are the most common, affecting more than 500 patients and 20 families respectively. Mutations in these genes may affect adipocyte differentiation, cell membrane integrity, DNA repair, lipid droplet formation, and lipolysis (Chen et al., 2022; Kountouri et al., 2023).

FPLD6 is caused by mutations in the lipase E, hormone-sensitive type (*LIPE*) gene, which encodes hormone-sensitive lipase (HSL). In adipose tissue, it primarily hydrolyzes stored triglycerides into free fatty acids, while in steroidogenic tissues, it converts cholesteryl esters into free cholesterol for steroid hormone production. The clinical symptoms of FPLD6 often present in adulthood, including atrophy of fat in the buttocks, hips, and lower limbs, accompanied by abnormal fat deposition in the neck, supraclavicular area, armpits, triceps and below, back, abdomen, and labia; multiple lipomas; and progressive myopathy. Muscle atrophy, elevated levels of creatine kinase, hypertriglyceridemia, DM, hypertension, and fatty liver disease can also manifest (Garenc et al., 2009; Fernandez-Pombo et al., 2021). FPLD6 was first reported in an Old Order Amish family by Albert et al. (2014). To date, there have been no more than 10 cases of FPLD6 reported worldwide, including three families from different countries and three unrelated patients with *LIPE* gene mutations causing multiple symmetric lipomatosis and/or FPLD (Albert et al., 2014; Farhan et al., 2014; Zolotov et al., 2017; Sollier et al., 2021).

Here, we report a case of FPLD6 in a young female patient carrying compound heterozygous mutations in the *LIPE* gene. This disease had not yet been reported in China, making this the first reported case.

## 2 Case presentation

A 20-year-old female patient, with a height of 1.6 m, weight of 50 kg, and body mass index of 19.53 kg/m^2^, was admitted to our department on 17 April 2023, due to “discovered hepatomegaly occurring for 1 month.” Upon physical examination, it was found that the liver was enlarged, extending approximately 12 cm below the ribs, and firm, with no tenderness upon touch. The spleen was not palpable, and there was an excess amount of fat in the neck and back areas. Upon further inquiry into the patient’s history, it was ascertained that she had been gaining weight since the age of 10, with a body type leaning towards obesity and weighing up to 65 kg at her heaviest. In the past 3 years, she lost 15 kg, with a noticeable reduction of fat in her limbs and abdomen, increased fat accumulation in her neck and back, and significant decrease in lower limb strength, and found it challenging to climb four flights of stairs. She denied having a history of smoking or drinking alcohol. She started menstruating at age 15, with irregular cycles, sometimes once a month, sometimes once in 3–4 months. Her parents were not closely related by blood. They were in good health and denied any history of muscle atrophy, fat redistribution, diabetes, or fatty liver. Her maternal grandfather and paternal grandmother had a history of DM. She had a 3-year history of DM and was treated with insulin. No dietary restrictions applied with little exercise. On 20 March 2023, she was admitted to our endocrinology department due to intermittent blurry vision. A glucose tolerance test indicated that she had type 2 DM (see Supplementary Table S1 for more details). She had been treated with subcutaneous injections of dulaglutide, NovoRapid^®^, and Lantus^®^; as well as oral hypoglycemic agents, including metformin, pioglitazone, Januvia™, and linagliptin; and lipid-lowering treatments, including fenofibrate and orlistat. Upon admission, lab tests showed significant improvements in her blood lipid and blood glucose levels compared to previous readings (Table 1). Hepatitis virus tests were negative, and immunological indices including antinuclear antibodies were normal. Among the four indicators of liver fibrosis, the Type IV collagen level increased to 52.65 ng/mL (normal value < 30 ng/mL). The alanine transaminase (170 U/L), aspartate aminotransferase (96 U/L), gamma-glutamyl transferase (127 U/L), urine microalbumin/creatinine ratio (569.5 mg/g), tubular function transferrin (0.69 mg/dL), and urine microalbumin (15.8 mg/dL) levels were all elevated. The 24-h urine protein was 0.27 g/d. The creatinine, urea nitrogen, and cystatin C levels were normal. The six sex hormones, adrenocorticotropic hormone, and cortisol levels were all normal. IGF-1 and type 1 DM autoantibodies were also within normal range. Considering the patient’s medical history, inherited metabolic diseases could not be ruled out. Further examinations, such as liver imaging, pathology, and high-throughput sequencing, were recommended.

**TABLE 1:** Levels of liver function, lipid metabolism, and blood glucose before and after treatment.

	Value (2023.3.21)	Value (2023.4.18)	Normal range
ALT (U/L)	170	28	0–40
AST (U/L)	96	39	5–34
ALP(U/L)	37	25.6	40–150
GGT (U/L)	127	53	9–64
LDH(U/L)	229	109	103–227
MAO(U/L)	Undetectable	5.43	<12
Triglyceride (mmol/L)	15.36	4.76	0.4–1.69
Cholesterol (mmol/L)	5.57	5.27	3.36–5.69
HDL (mmol/L)	0.45	0.64	1.04–1.83
LDL (mmol/L)	0.83	3.2	<3.3
HbA1c (%)	12	8.9	4.8–6.0
FBG (mmol/L)	13.61	7.23	3.9–6.11

ALT, alanine transaminase; AST, aspartate aminotransferase; ALP, alkaline phosphatase; GGT, gamma-glutamyl transferase; LDH, lactic dehydrogenase; MAO, monoamine oxidase; HDL, high density lipoprotein; LDL, low density lipoprotein; HbA1c, glycosylated hemoglobin; FBG, fasting blood glucose.

**FIGURE 1:**
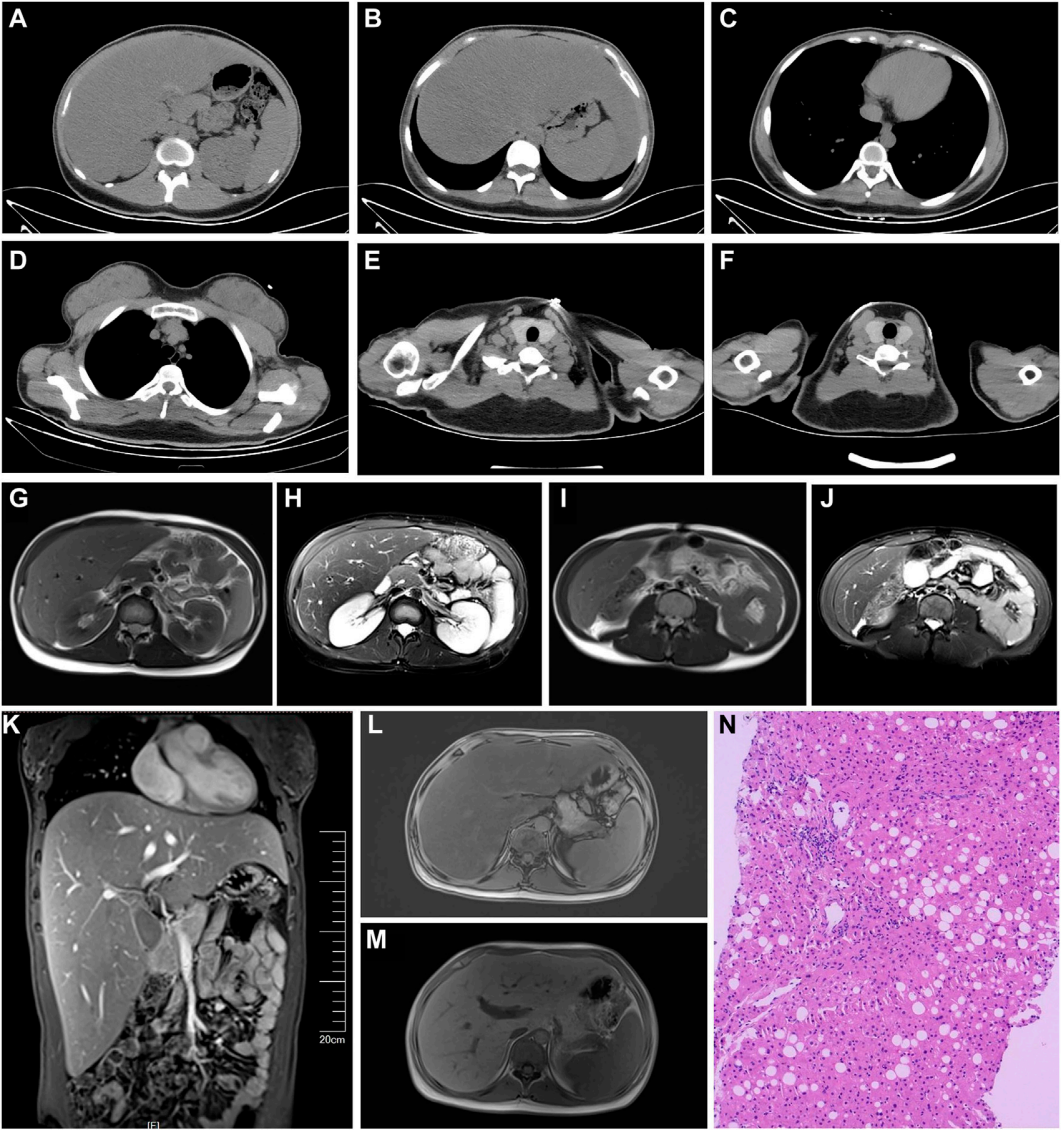
**(A–J)** Computed tomography scans. **(A)** Thin abdominal wall: fat and thick back fat, fatty liver (Ratio of liver CT value to spleen CT value = 41/54 = 0.76). **(B)** Decreased intermuscular fat in the abdominal wall and psoas major muscle. **(C)** Thin chest fat and decreased intermuscular fat. **(D)** Thin fat on the chest wall, relatively thick fat on the back, and very little fat in the armpit. **(E)** Noticeably thicker fat in the posterior neck area. **(F)** Lot of fat in the neck area. **(G–M)** Magnetic resonance imaging. **(G–J)** Thin abdominal fat layer and decreased intermuscular fat. **(M)** Significantly enlarged liver. **(L,M)** Fatty liver. **(N)** Liver pathology (Hematoxylin and Eosin stain, ×200): The structure of hepatic lobules is preserved in the liver tissue, with a small amount of infiltrated lymphocytes and an infiltration of a small number of plasma cells infiltration in the portal area. The interlobular bile duct structure is present. There is an enlarged fibrosis, with observable fibrous tissue septa. Partial hepatic cells have undergone fatty degeneration, predominantly in the form of large vesicles. The Steatosis, Activity, and Fibrosis score (steatosis 2, ballooning 1, lobular inflammation 2, fibrosis 2) was consistent with non-alcoholic steatohepatitis (equivalent to G1-2/S2).

Computed tomography scans (Figures 1A–J) showed fatty liver. The overall distribution of body fat appeared to be less in the front and more in the back. There was less fat in the chest wall and abdomen, more in the back, and very little in the armpits; the fat in the posterior neck area was noticeably thicker; the intermuscular fat in the chest, abdominal wall, and psoas major muscle was reduced. Magnetic resonance imaging scans (Figures 1G–M) showed hepatomegaly and fatty liver. The abdominal fat layer was thin, and the intermuscular fat was reduced. The liver elasticity hardness was 9.5 kPa and fat attenuation 269 dB/m. The liver tissue pathology (Figure 1N) showed Moderate fatty liver. The Steatosis, Activity, and Fibrosis score (steatosis 2, ballooning 1, lobular inflammation 2, fibrosis 2) was consistent with non-alcoholic steatohepatitis (equivalent to G1-2/S2). And electromyogram showed peripheral nerve lesions on both lower limbs. The motor nerve conduction velocity of the bilateral common peroneal nerves had slowed down, with a normal compound muscle action potential amplitude, and the sensory conduction velocity of the right superficial peroneal nerve had slowed down, with a normal sensory nerve action potential amplitude. Bilateral fundus photography (Supplementary Figure S1) revealed that within the visible range, the optic disc boundary was clear and of normal color; the cup-to-disc (C/D) ratio was normal; the shape of the retinal blood vessels was acceptable; the foveal reflex was positive, and there were multiple spots of hemorrhage and exudation visible in the posterior pole. Arterial stiffness was recorded as right 1,150 baPWV, left 1,156 baPWV, which was slightly stiffer compared with that of a healthy 20-year-old female. An ultrasound of the urinary system, cardiac ultrasound, and carotid ultrasound showed no abnormalities.

High-throughput sequencing was performed using genomic DNA extracted from peripheral blood leukocytes for sequencing analysis using the Blood Genomic DNA Miniprep Kit (Axygen, Silicon Valley, United States). Specifically for the proband, targeted next-generation sequencing was performed to capture disease-related genes. The genes were enriched using biotinylated capture probes, and the capture experiment was performed following the manufacturer’s protocol (MyGenostics, Beijing, China), as previously described (Hollysz et al., 2011). Subsequently, the samples were sequenced on the Illumina NextSeq 500 platform (San Diego, California, United States) with a coverage of ×50. Variant calling was conducted using Illumina NextSeq Reporter Software, with the NCBI37/hg19 assembly of the human genome serving as the reference sequences. Sanger sequencing confirmed the *LIPE* mutation in the patient’s parents.

**FIGURE 2:**
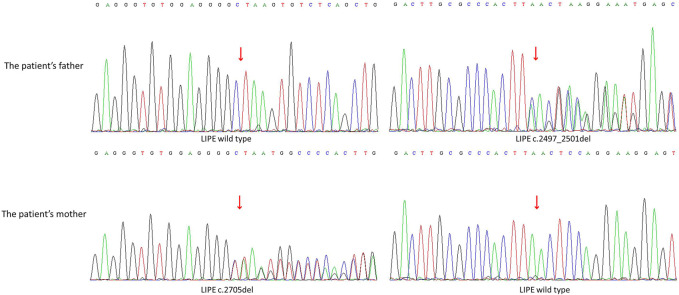
Patient’s parental generation sequencing verification analysis. Compound heterozygous mutations was analyzed in the *LIPE* gene in the sample: A frameshift mutation c.2497_250ldel in the exon8 results in an amino acid change p. Glu833LysfsTer22. The father has a heterozygous mutation at this locus, while the mother shows no mutation at this locus; A frameshift mutation c.2705del in the exon9 results in an amino acid change p. Ser902ThrfsTer27. The mother has a heterozygous mutation at this locus, while the father shows no mutation at this locus.

Two heterozygous mutations were analyzed in the *LIPE* gene of the sample. A frameshift mutation c.2497_250ldel in the exon8 results in an amino acid change p. Glu833LysfsTer22. A familial verification analysis revealed that the patient’s father had a heterozygous mutation at this locus, while the mother showed no mutation at this locus. A frameshift mutation c.2705del in the exon9 of the *LIPE* gene results in an amino acid change p. Ser902ThrfsTer27. After familial verification analysis, it was determined that the patient’s mother had a heterozygous mutation at this locus, while the father showed no mutation at this locus (Figure 2). According to the Criteria for Classifying Pathogenic Variants from the American College of Medical Genetics and Genomics guidelines, the two variations were initially determined to be pathogenic [PVS1+PM2_Supporting + PM3 (Trans)].

Also identified from the sample was a hotspot mutation (C>T) at position chrM:1494 in the MT-RNR1 gene, with a mutation base/(mutation base + reference base) (mutation frequency) of 1292845/(1292845 + 29975) (97.73%), which is a pathogenic mutation leading to aminoglycoside-induced deafness. After familial verification analysis, the mutation frequency at this locus in the patient’s mother was 941497/(941497 + 73050) (92.79%).

The patient was ultimately diagnosed with FPLD6.

## 3 Discussion

Reported cases of FPLD6 have shown that patients range in age from 36 to 76 years, are mostly female, and experience varied clinical manifestations. All patients experienced fat redistribution and DM, with hypertriglyceridemia, fatty liver, and hypercreatine kinase-emia also being common. Fat redistribution most often occurs in middle age. Our patient was only 20 years old, being the youngest case reported so far. At the age of 17, fat redistribution occurred, with overall fat reduction in the anterior part of the body, including the chest wall, abdomen, and limbs; fat accumulation in the neck and back; and metabolic abnormalities such as DM, hypertriglyceridemia, fatty liver, and hypercreatine kinase-emia. The liver was significantly enlarged, and pathology revealed moderate hepatic steatosis with fibrosis. The elevation of type IV collagen in the lab tests also confirmed this. The patient had already exhibited muscle weakness, and electromyography indicated peripheral nerve damage in both lower limbs. Muscle damage was also reported in three previously reported cases, but peripheral nerve damage was not mentioned (Farhan et al., 2014; Zolotov et al., 2017). The neuropathy manifested in other types of FPLD is considered to be caused by diabetes (Kountouri et al., 2023). When lipodystrophic syndrome presents with neurological damage, it is important to distinguish it from Multiple Symmetric Lipomatosis (MSL). Patients with MFN2-associated MSL exhibit a Charcot–Marie–Tooth neuropathy, which manifests as a four-limb peripheral sensitive-motor axonal neuropathy of early onset (Bennett et al., 2008). Our patient presented with blurred vision, and examination revealed retinal hemorrhage and exudates. Sollier et al. (2021) also reported a significant number of retinal deposits in three patients. Hypertension also occurs in some FPLD6 patients. Although our patient’s blood pressure, cardiac ultrasound, and carotid ultrasound were normal, arterial stiffness increased compared to individuals of the same age. In addition, both urine microalbumin/creatinine and urinary transferrin levels were elevated, indicating tubular kidney damage, which had not yet been reported in FPLD6 patients. According to a multi-center study in Turkey, Renal biopsies in 9 patients with generalized or partial lipodystrophy (mainly FPLD2 or FLPD3) revealed podocyte injury, focal segmental sclerosis, thickening of glomerular basal membranes, mesangial matrix and tubular abnormalities at various levels (Akinci et al., 2018).

FPLD6 occurs due to variations in the *LIPE* gene. The *LIPE* gene, which is located on chromosome 19q13.2, is composed of 15 exons (Hollysz et al., 2011). The polymorphism in this region (LIPE-60 C>G) is associated with hepatic steatosis, obesity, DM, and dyslipidemia (Hsiao et al., 2013). The HSL encoded by the *LIPE* gene is a key enzyme in lipid metabolism that is involved in the hydrolysis of intracellular triglycerides and the release of fatty acids as an energy source for adipose tissue (Kulcenty et al., 2015). In obesity, the rate of fat breakdown decreases as HSL levels decrease (Stelmanska et al., 2015). The Arg611Cys variation in the *LIPE* gene leads to impaired lipolytic capacity, which affects glucose homeostasis and the risk of developing type 2 DM (Muller et al., 2022). The above can explain the reason for metabolic abnormalities in patients with FPLD6. HSL/*LIPE* is also expressed in hepatic stellate cells and possesses retinyl ester hydrolase activity, thus playing a role in the metabolism of vitamin A within the liver (Shajari et al., 2019). Yuksel et al. (2023) reported that Lipe^−/−^ mice, which were created using CRISPR-Cas9 technology, exhibited an accumulation of subretinal microglial cells, retinal degeneration, and the concurrent decline in visual function. Initially, we believed that the patient’s retinal changes were due to diabetes, but the researches indicated that this might also directly result from mutations in *LIPE*, along with factors related to vitamin A deficiency.

Currently, there are no standardized criteria for the diagnosis of FPLD. Diagnosis is primarily based on medical history, physical examination, body composition, and assessment of metabolic complications, with confirmatory genetic analysis being the most crucial assessment. Recently, French researchers characterized the diagnostic pathway of genetic lipodystrophies patients through a cross-sectional study conducted using a self-reported patient questionnaire. A “morphotype change,” which includes either lipohypertrophy or lipoatrophy, is the most common initial symptom. The most commonly reported combination of initial symptoms involves facio-cervical lipohypertrophy along with muscular hypertrophy in FPLD. While techniques like dual-energy X-ray absorptiometry can be beneficial for body composition analysis, clinical examinations that encompass waist and hip measurements, biacromial diameter, and skinfold assessments serve to refine the diagnosis of lipodystrophy. Given that alterations in physical appearance may be subtle, requesting photographs from patients at different stages of life can be immensely beneficial. This is especially pertinent for FPLD, as changes in physical appearance tend to manifest progressively around the time of puberty (Mosbah et al., 2024). It has been reported that patients with *LIPE* gene mutations are often homozygous (see Table 2). The parents of our patient were not closely related by blood, and genetic analysis revealed that the patient had compound heterozygous mutations in the *LIPE* gene: c.2497_2501del (p.Glu833LysfsTer22) and c.2705del (p.Ser902ThrfsTer27) heterozygous mutations. This was newly discovered compound heterozygous mutations in the *LIPE* gene that was determined to cause FPLD6. Congenital genetic disease was considered. Additionally, our patient was found to have a hotspot mutation at chrM:1494 in the mitochondrial MT-RNR1 gene, which can lead to aminoglycoside-induced deafness. Therefore, the patient should avoid the use of aminoglycoside medications.

**TABLE 2:** Cases of FPLD6 caused by LIPE mutations.

Author	Parental consanguinity	Amino acid change	Type of mutation	Effect	Homozygous/heterozygous
Albert	yes	p.V767Gfs*102	c.2300_2318del	Frameshift	Homozygous
Farhan	yes	p.Ala507fs*563	c.ins1519CG	Frameshift	Homozygous
Zolotov	yes	p.Glu1035*	c.3103G>T	Nonsense	Homozygous
Sollier	yes	p.Glu943Glyfs*22	c.2828del	Frameshift	Homozygous
	no	p.Leu631Glyfs*57; p.Arg693Valfs*76	c.1890_1891del; c.2077del	Two frameshift	Heterozygous
	no	p.Gln421*	c.1261C>T	Nonsense	Homozygous
Our patient	no	p.Glu833Lysfs*22; p.Ser902Thrfs*27	c.2497_2501del; c.2705del	Two frameshift	Compound heterozygous

Currently, there is no cure for FPLD, so diet and physical exercise are fundamental approaches for managing FPLD. The recommended diet includes 50%–60% carbohydrates, 20%–30% fats, and approximately 20% protein (Hussain and Garg, 2016). Physical exercise can help improve metabolism, but an individual cardiac assessment is necessary. Patients who are prone to arrhythmias or cardiomyopathy should avoid intense exercise (Fernandez-Pombo et al., 2023). Patients with IR and DM should receive standard treatment, including oral medications (metformin was a first-line medication) and insulin. However, studies have indicated that metformin may actually heighten insulin resistance in a patient with SHORT syndrome, but additional research is necessary to validate these results. Many patients require concentrated forms of insulin (Lambadiari et al., 2021; Iizaka et al., 2023). Statins remain the preferred therapy for dyslipidemia. For severe hypertriglyceridemia (>500 mg/dL), fibrates and long-chain omega-3 fatty acids should be added, although caution is advised for patients with myasthenia or muscle atrophy (Sollier et al., 2020). Volanesorsen reduces serum triglyceride levels and improves the metabolic status of FPLD patients by decreasing the synthesis of apolipoprotein C-III in the liver (Oral et al., 2022). In our case, the patient achieved significant control over blood glucose levels and a marked decrease in triglyceride levels after 1 month of treatment with oral medications, insulin, fenofibrate, and dulaglutide. Metreleptin, a recombinant analog of human leptin, has been approved by the European Medicines Agency (EMA), not only for generalized lipodystrophy but also for partial lipodystrophy patients >12 years of age when standard treatments have failed. (https://www.ema.europa.eu/en/medicines/human/EPAR/myalepta). It can improve hypertriglyceridemia, glycated hemoglobin, insulin sensitivity, and liver volume in patients with FPLD. Its tolerability is good, with the most common adverse reactions being abdominal pain, hypoglycemia, and nausea (Chou and Perry, 2013; Oral et al., 2019). Research has shown that the use of metreleptin for 12 months in patients with FPLD2 and FPLD3 resulted in a reduction in triglycerides and HbA1c levels (Sekizkardes et al., 2019). In some NASH patients with dysregulated fat metabolism, metreleptin treatment can reduce liver fat deposition and damage (Akinci et al., 2021). Regrettably, this medication has not yet been launched in China, and the patient has no access to it.

The current study has some limitations. This is a single case report, limiting generalizability of the findings. Since there are no additional blood samples, functional validation of the identified LIPE mutations is not performed.

## 4 Conclusion

Here, we reported a rare case of FPLD6 caused by novel compound heterozygous mutations in the *LIPE* gene. In addition to the classic symptoms of hypertriglyceridemia, DM, and liver fat metabolism disorder, this young patient exhibited retinal changes, peripheral nerve damage, and renal tubular injury. When these manifestations appear, FPLD should be considered as a possible differential diagnosis. This condition requires a balanced diet and moderate exercise. The treatment of metabolic complications necessitates conventional antidiabetic or lipid-lowering medications. Where possible, the use of the specific medication metreleptin may be considered.

## Data Availability

The raw data supporting the conclusions of this article will be made available by the authors, without undue reservation.
