# Supplementary material for: Case report: First Chinese patient with family partial lipodystrophy type 6 due to novel compound heterozygous mutations in the LIPE gene
Source: Front Genet. 2024 Jul 24;15:1417613. doi: 10.3389/fgene.2024.1417613 (PMC11303181; doi:10.3389/fgene.2024.1417613)
Supplement: Supplementary file 1 [file Table1.DOCX]

# Supplement table

Supplement table1 Insulin release test

|  | 0min | 30min | 60min | 120min | 180min |
| --- | --- | --- | --- | --- | --- |
| Blood glucose(mol/L) | 13.61 | 14.68 | 18.6 | 22 | 19.8 |
| C-peptide(ng/mL) | 2.57 | 2.78 | 3.82 | 3.78 | 4.48 |
