# Supplementary material for: Case report: First Chinese patient with family partial lipodystrophy type 6 due to novel compound heterozygous mutations in the LIPE gene
Source: Front Genet. 2024 Jul 24;15:1417613. doi: 10.3389/fgene.2024.1417613 (PMC11303181; doi:10.3389/fgene.2024.1417613)
Supplement: Supplementary file 2 [file Image1.PDF]

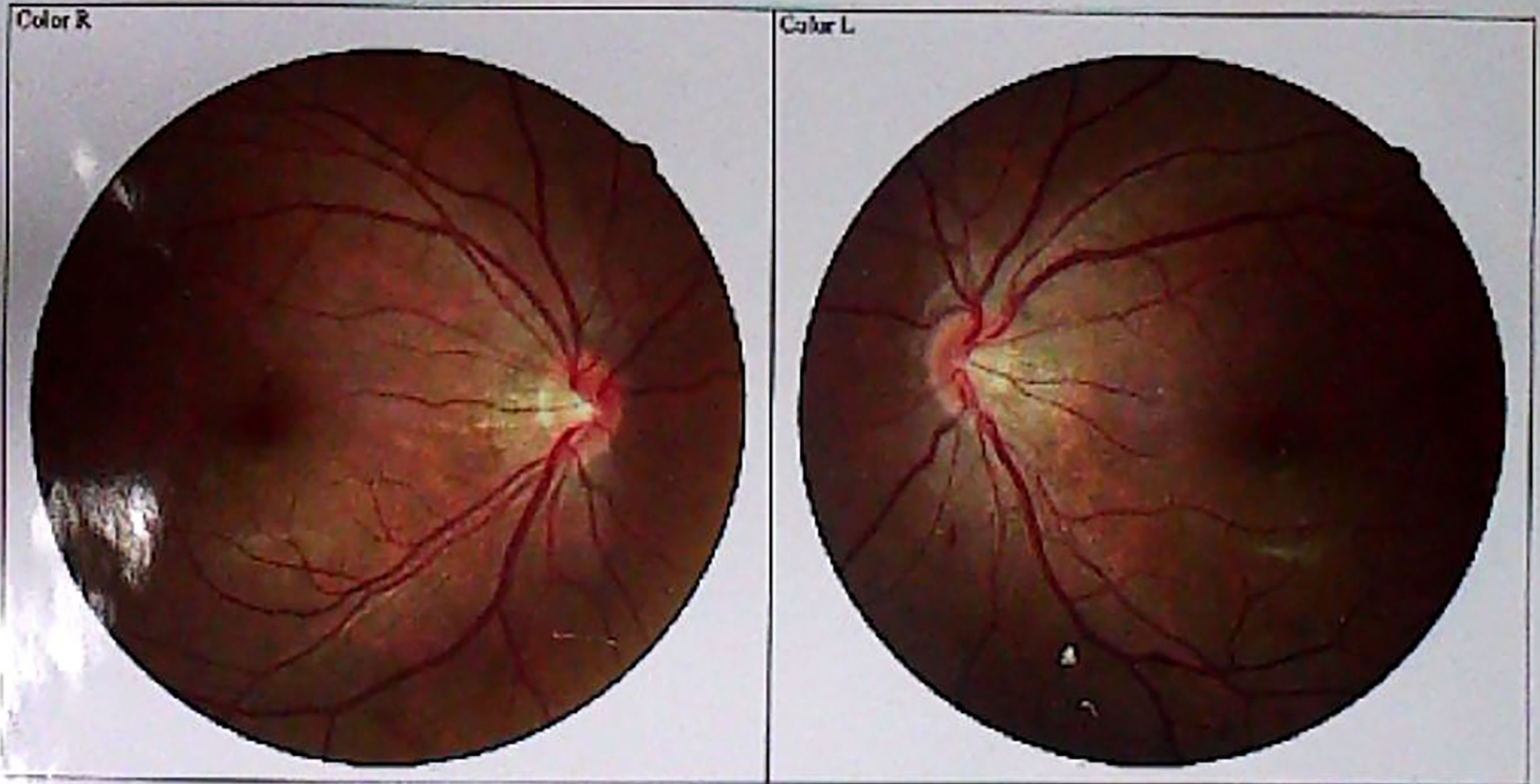
Color R
Color L

## Supplement Figure Legends

**Supplemental Figure 1.** Fundus photography: Within the visible range, the optic disc boundary is clear and of normal color, the cup-to-disk (C/D) ratio is normal; the shape of the retinal blood vessels is acceptable; the foveal reflex is positive; and there are multiple spots of hemorrhage and exudation visible in the posterior pole.
